# Predictive factors of successful microdissection testicular sperm extraction

**DOI:** 10.1186/2051-4190-23-5

**Published:** 2013-10-02

**Authors:** Aaron M Bernie, Ranjith Ramasamy, Peter N Schlegel

**Affiliations:** Department of Urology, Weill Cornell Medical College, New York-Presbyterian Hospital, New York, NY USA

**Keywords:** Sperm retrieval, Testicular sperm extraction, Non-obstructive azoospermia, TESE, récupération de spermatozoïdes, extraction de spermatozoïdes testiculaires, azoospermie non obstructive, TESE

## Abstract

Azoospermia in men requires microsurgical reconstruction or a procedure for sperm retrieval with assisted reproduction to allow fertility. While the chance of successful retrieval of sperm in men with obstructive azoospermia approaches >90%, the chances of sperm retrieval in men with non-obstructive azoospermia (NOA) are not as high. Conventional procedures such as fine needle aspiration of the testis, testicular biopsy and testicular sperm extraction are successful in 20-45% of men with NOA. With microdissection testicular sperm extraction (micro-TESE), the chance of successful retrieval can be up to 60%. Despite this increased success, the ability to counsel patients preoperatively on their probability of successful sperm retrieval has remained challenging. A combination of variables such as age, serum FSH and inhibin B levels, testicular size, genetic analysis, history of Klinefelter syndrome, history of cryptorchidism or varicocele and histopathology on diagnostic biopsy have provided some insight into the chance of successful sperm retrieval in men with NOA. The goal of this review was to evaluate the preoperative factors that are currently available to predict the outcome for success with micro-TESE.

## Résumé

Pour permettre une fécondité chez l’homme, l’azoospermie nécessite une reconstruction par microchirurgie ou une procédure de récupération de spermatozoïdes avec assistance médicale à la procréation. Alors que les chances d’une extraction positive de spermatozoïdes chez les hommes qui présentent une azoospermie obstructive atteignent plus de 90%, ces chances ne sont pas aussi grandes chez les hommes qui ont une azoospermie non obstructive (NOA). Les procédures conventionnelles telles que l’aspiration à l’aiguille fine du testicule, la biopsie testiculaire et l’extraction testiculaire de spermatozoïdes sont couronnées de succès chez 20-45% des hommes avec NOA. En cas d’extraction de spermatozoïdes testiculaires par microdissection (micro-TESE), les chances d’un prélèvement positif peuvent aller jusqu’à 60%. Malgré cette augmentation des chances, la possibilité d’informer les patients avant l’intervention de leurs chances d’avoir un prélèvement de spermatozoïdes positif reste un défi. La combinaison de variables telles l’âge, les taux sériques de FSH et d’inhibine B, le volume testiculaire, les analyses génétiques, un antécédent de syndrome de Klinefelter, de cryptorchidie ou de varicocèle, et l’histopathologie du tissu recueilli lors d’une biopsie diagnostique, a fourni un aperçu des chances d’obtenir un prélèvement positif de spermatozoïdes chez les hommes avec NOA. L’objectif de cette revue est d’évaluer les facteurs préopératoires qui sont actuellement disponibles pour prédire une issue positive à une micro-TESE.

## Introduction

Men undergoing evaluation for infertility are found to have azoospermia, or lack of sperm in the ejaculate, up to 10% of the time [1]. Approximately 60% of these cases are due to non-obstructive azoospermia (NOA) [2] a condition in which men have impaired production of sperm. Men with NOA require some form of sperm retrieval procedure in conjunction with intra-cytoplasmic sperm injection (ICSI) [3] to father their own children.

Microdissection testicular sperm extraction (Micro-TESE), currently one of the most popular sperm retrieval procedures for men with NOA, was first described in 1999. Micro-TESE provides the advantage of allowing the surgeon to selectively identify seminiferous tubules most likely to contain spermatozoa based on the larger and more opaque appearance of those tubules. With micro-TESE, successful sperm retrieval has been reported in men up to 63% of men [4], whereas conventional and more limited sperm retrieval procedures have reported success rates from 20% (percutaneous testicular biopsies) [5] to 45% (open testis biopsies) [6]. Studies formally comparing conventional testicular sperm extraction (TESE) vs. micro-TESE have seen similar results, with sperm retrieval rates significantly higher when the procedure is performed with a microsurgical approach [7, 8].

The technique for performing micro-TESE was originally described by Schlegel [6]. The procedure is initially performed under 6-8x magnification to optimize visualization of blood vessels and allow for a wide incision in the tunica albuginea in an avascular plane. Next, the magnification is increased to 15-20x for identification of larger individual seminiferous tubules that are more opaque than other surrounding tubules. These tubules are then cut into small pieces to release spermatozoa from the tubules. Finally, this processed sample is examined for viable spermatozoa [8].

While the success of micro-TESE compared to other sperm retrieval techniques has been widely accepted, a full understanding of predicting preoperatively whom the procedure is going to be successful is not entirely clear and remains controversial [9]. Several studies have analyzed preoperative variables used to predict sperm retrieval with conventional procedures [10–13]. In this review, we will evaluate preoperative variables such as age, FSH, testicular volume, inhibin B, genetics, Klinefelter syndrome, history of varicocele, cryptorchidism, as well as intraoperative variables such as histopathology and tubular diameter and their relevance for predicting the outcome of micro-TESE. These variables were determined by reviewing the available literature on prediction of success in sperm retrieval techniques, with a focus on those reviews that are dedicated to micro-TESE. Table 1 summarizes all of these factors and their role in prediction of sperm retrieval during micro-TESE.

**Table 1:** **Overview of preoperative factors considered for prediction of micro-TESE outcom**e

Predictive factors of micro-TESE	Comments
**Serum FSH**	FSH, although a good predictor of global testicular function, does not serve by itself as a predictor of successful micro-TESE, but models have shown some predictive value when used in conjunction with other variables [14, 19–22]
**Serum Inhibin B**	Inhibin B, like FSH, does not serve as a good predictor of micro-TESE by itself, but models have shown predictive value when used in conjunction with other variables [15, 19]
**Histopathology**	Histopathology is likely the greatest single predictor of successful micro-TESE, but the requirement of a separate surgical procedure for diagnosis makes its role very limited [35, 59]
**Testicular volume**	The data on testicular volume and its predictive value for micro-TESE is limited, and suggests that it is not a good predictive variable for micro-TESE [36, 37]
**Genetics**	Genetics, particularly Y chromosome microdeletions, are very helpful in predicting success of micro-TESE; men with AZFc microdeletions have very good chance of successful micro-TESE, whereas those men with AZFa or AZFb have a low probability of success [6, 39]
**Klinefelter’s Syndrome (KS)**	Men with KS have successful micro-TESE rates similar to or better than all men with NOA, and KS itself is a good prognostic factor for sperm retrieval [43, 45]
**Age**	While advanced paternal age may play a role in decreased pregnancy rates, the limited studies thus far show that it does not play a predictive role for micro-TESE (unpublished data)
**Cryptorchidism**	Men with a history of cryptorchidism have successful micro-TESE rates to men without cryptorchidism, suggesting that it does not play a predictive role in the success of micro-TESE [50]
**Varicocele**	The need for varicocelectomy in men with a varicocele and NOA prior to micro-TESE is debated, but men with a clinical varicocele who undergo varicocelectomy prior to micro-TESE have higher sperm retrieval rates compared to men with all other causes of NOA, suggesting that varicocele repair is a positive prognostic factor for men undergoing micro-TESE [55, 57, 58]

### Follicle-stimulating hormone (FSH)

Follicle-stimulating hormone (FSH) is a glycoprotein produced in the pituitary gland after stimulation by GnRH from the hypothalamus. It exerts its action by binding to receptors on Sertoli cells in the testes leading to production of hormones (eg. inhibin and activin) and nutrients required for germ cell maturation [14]. Because of this mechanism of action, many have theorized that FSH may be useful in predicting the outcome of sperm retrieval procedures, in that patients with very high levels of FSH would tend to have a global failure of sperm production within the testis.

FSH levels differ significantly in patients with whom sperm is retrieved vs. patients where sperm is not retrieved with conventional TESE [15, 16], and azoospermic men with high FSH have been shown to have lower sperm retrieval rates [17]. Some have advocated that exogenous administration of recombinant FSH can increase the success of sperm retrieval when such treatment is given prior to a sperm retrieval procedure [18]. In a study by Aydos et al. 108 men with NOA and normal FSH levels were included in the study. The study population received pure FSH three times per week for three months (vs control group that received no treatment) followed by micro-TESE. Men who received therapy with pure FSH were almost twice as likely to have sperm retrieved during their procedure (64% vs. 33% sperm retrieval rates in the treatment group compared to the control group) despite a lack of change in FSH levels for treated men. Tsujimura et al. have reported that preoperative serum FSH level in combination with other factors may help predict the success of micro-TESE [19]. These findings would suggest a predictive role of serum FSH is used to project the outcome of sperm retrieval, and suggest a potential therapeutic role for increasing sperm production prior to performing a procedure.

Despite these outlier studies, a majority of evaluations have shown that the predictive value of FSH for success of TESE and other sperm retrieval methods is either low or non-existent [20–23]. With regards to micro-TESE, we have previously shown that men with high FSH have similar or better sperm retrieval compared to those with lower FSH [24, 25]. In fact, a subset of men with normal FSH levels could have a uniform histological pattern of maturation arrest and extremely poor sperm retrieval rates [26]. These apparent discrepancies can be explained by the fact that FSH serves as an indicator of overall testicular function, as suggested by previous studies showing the connection between elevated FSH levels and poor histological features and lower sperm retrieval rates with testicular biopsy [24, 27]. So, while FSH is believed to be related to the number of germ cells present in the testicle, a high FSH level may not reflect the relatively few seminiferous tubules that could contain sperm [4, 28]. Because micro-TESE allows the surgeon to find areas of advanced spermatogenesis in the testicle, successful sperm retrieval is still possible in men with high FSH. High and abnormal FSH levels should not be considered a contraindication for micro-TESE in experienced hands.

### Inhibin B

Inhibin B is a glycoprotein hormone produced by Sertoli cells in the testicle. It acts as a negative regulatory factor on FSH and subsequently is found to be low in men with testicular dysfunction and low levels of spermatogenesis [29]. Because of this, inhibin B has been found to correlate well with the degree of spermatogenesis seen on histologic analysis that occurs in the testicle [30]. Similar to the findings seen with FSH, there is a significant difference in the level of inhibin B seen in men who successfully undergo conventional TESE vs. those that do not have sperm retrieved [15].

Because of these findings, it was thought that inhibin B would prove to be a valuable marker for success of sperm retrieval. Several studies have shown that inhibin B may have a role in providing an accurate prediction with respect to sperm retrieval [31, 32]. Bohring et al. reported a sensitivity and specificity approaching 80% for inhibin B with respect to prediction of histologic features in the testicle, although the sensitivity and specificity of this marker both dropped to less than 50% when attempting to predict sperm retrieval [31]. Ballesca et al. found that inhibin B levels were predictive of TESE outcome with a sensitivity of 90% and specificity of 100% when comparing men with nonobstructive azoospermia to those with either obstructive azoospermia or normal semen analysis [32]. Similar to FSH, inhibin B has also been found to be a predictive factor for outcome of micro-TESE, but only when combined with other variables in predictive models of micro-TESE [19].

Despite these findings, a majority of studies refute the notion that inhibin B is a predictive marker at all [10, 20, 33, 34]. Tunc et al. found no difference in inhibin B levels in patients with a successful vs. unsuccessful TESE procedure, with specificity as low as 14% [10]. Vernaeve et al. found similar results, once again with no difference in inhibin B levels in men with a successful vs. unsuccessful TESE procedure.

These contradictory findings have made the role of inhibin B as a predictive marker controversial, but the majority of findings suggest that it remains a poor predictive marker for micro-TESE outcome. In conjunction with other preoperative markers it could have a small predictive value, but we do not utilize it as a routine evaluation tool in men with NOA prior to micro-TESE.

### Testis volume

Larger testes have often been considered a sign of normal spermatogenesis. Small and atrophic testicles are seen in many men with nonobstructive azoospermia. Schoor et al. defined that men with a testicular long axis of 4.6 cm or less as well as an FSH of 7.6 mIU/mL or less are likely to have nonobstructive azoospermia based on these factors alone [35].

While this may be the case, the use of testicular size alone to predict outcome is very limited. While there has been shown to be a positive correlation between testicular volume and both success in conventional TESE and sperm aspiration [15] as well as micro-TESE [19], it has not been consistently found to be a good predictive variable [36]. In our analysis, testicular volume played no role in the ability to successfully predict outcome of micro-TESE [37]. Similar to FSH, while small testicles may represent a global pattern of poor spermatogenesis, testis volume may not predict the relatively rate tubules with spermatogenesis that could be identified with micro-TESE. Testis size still remains to be a poor predictor of successful sperm retrieval outcome [37].

### Genetics

Microdeletions that occur on the Y chromosome, specifically in the AZFa, AZFb and AZFc regions have been determined to provide valuable prognostic information about the chance of sperm retrieval. According to the American Urological Association guidelines, men presenting with infertility found to have nonobstructive azoospermia or severe oligospermia (<5 million/ml) should undergo karyotype and Y-chromosome microdeletion testing [38].

Microdeletions in AZFa and AZFb provide a worse prognosis, and no man has been reported to have retrievable sperm [39]. Isolated AZFa deletions, while rare, are associated with a Sertoli cell-only histologic pattern with no sperm [40], and AZFb deletions are most often seen in conjunction with at least focal maturation arrest [39]. Because of these observations, micro-TESE is contraindicated in patients found to have either complete AZFa or AZFb microdeletions. Patients with deletions in the AZFc region, the most common microdeletion seen, either have rare sperm in the ejaculate or are often able to have successful sperm retrieval with micro-TESE [6]. Stahl et al. examined 149 patients with microdeletions and found that the sperm retrieval rate was 71.4% in men with AZFc deletions, but that there was no sperm retrieved in any men with AZFa, AZFb, AZFb + c or complete Yq deletions [41].

The use of genetic testing for Y chromosome microdeletions in men with nonobstructive azoospermia remains critical, and should be routinely used prior to micro-TESE.

### Klinefelter syndrome

Klinefelter syndrome (KS) is the most common genetic abnormality seen in men with infertility. Men with non-mosaic KS are azoospermic, therefore not able to produce viable pregnancies without assisted reproductive technologies [42, 43]. Men with non-mosaic KS have a favorable success with micro-TESE. Several studies have evaluated preoperative factors such as testis volume, serum testosterone and human chorionic gonadotropin (hCG) [13, 44] to predict successful sperm retrieval in men with KS and have had varying results.

In our experience, there was no predictive value of serum FSH, LH or testis volume for sperm retrieval in patients with KS [45]. Sperm were successfully retrieved in 68% of this cohort, slightly greater than most reported retrieval rates with micro-TESE. Interestingly men with normal testosterone levels (>300 ng/dL) had up to 85% chance of successful sperm retrieval. In addition, men with who responded to preoperative therapy with clomiphene citrate, aromatase inhibitors, HCG or a combination of these hormonal interventions with increased serum testosterone levels to ≥ 250 ng/dL had a higher sperm retrieval rate (SRR) than men who did not respond.

### Age

Advanced paternal age has been considered as a possible factor that could contribute to the success of sperm retrieval procedures, with the suggestion that as men age, the areas of active spermatogenesis would decrease. It is not known whether older men have more azoospermia than younger men. Studies on paternal age and its effect on fertility are limited and no specific male cut-off age where fertility is negatively affected has been defined [46, 47]. Despite this, there has been a suggestion that advanced paternal age leads to lower pregnancy rates and live birth rates, at least when men are age 45-50 or older [48, 49]. We found no association between male age and outcome of micro-TESE, and the overall sperm retrieval rate was actually highest in men 40 years or older and we found no upper limit for male age at which micro-TESE was successful (unpublished data).

### Cryptorchidism

We have previously shown that men with cryptorchidism had a good chance of sperm retrieval when undergoing micro-TESE [37, 50]. In fact, cryptorchid men tended to have slightly higher sperm retrieval rates (74%) relative to all other men with nonobstructive azoospermia (58%), although the difference in pregnancy rates was not statistically significant. In men with cryptorchidism, there was a correlation with both testicular volume and age at orchiopexy, whereas these associations were not seen in the entire cohort of men with nonosbtructive azoospermia. These results are similar to other series of men with cryptorchidism and nonobstructive azoospermia [51] including men who have undergone bilateral orchidopexy [52], suggesting that these men have a good chance of successful sperm recovery with micro-TESE.

### Varicocele

Varicoceles in men often lead to poor sperm quality as well as azoospermia [53]. For that reason, many men with a varicocele and azoospermia will undergo a varicocele repair prior to attempts at surgical sperm retrieval in the hopes of obtaining sperm in the ejaculate. Varicocelectomy for NOA however still remains controversial. Schlegel and Goldstein outlined several of the potential, but controversial indications for varicocele repair, including low testosterone, prevention of progressive testicular dysfunction, testicular pain and nonobstructive azoospermia [54]. There have been reports suggested increased sperm production in over half of patients leading to detectable sperm in the ejaculate after varicocelectomy in men with azoospermia [55], as well as reports of adequate sperm in the ejaculate after varicocelectomy in as few as 10% of patients [56]. Similarly, there have been both reports of improved sperm retrieval rates after varicocelectomy prior to micro-TESE in patients with nonobstructive azoospermia with a clinical varicocele [57], as well as reports suggesting that the varicocelectomy in these patients does not improve outcomes of micro-TESE [56]. Because of these conflicting results, the need for varicocelectomy in men prior to micro-TESE for sperm retrieval remains controversial. Haydardedeoglu et al compared men who had NOA and a clinical varicocele to men with NOA and no varicocele. They found that the men with a varicocele whom underwent varicocelectomy had higher rates of sperm retrieval compared to men with NOA and no varicocele [58], suggesting that the repair of a varicocele in men with NOA may play a predictive role in success of micro-TESE.

### Histopathology

Perhaps the strongest predictor of successful sperm retrieval is testicular histopathology. Many of the patients that are seen at a large tertiary referral center have had previous fertility counseling or procedures, and it is often helpful to include this data when counseling these patients on their chance of success with micro-TESE. For example, a patient may be seen who has had a testicular biopsy in the past, and because of this his histopathology is known. While many practitioners would not have done or recommended this procedure, the data is available and it is a useful piece of information that can be utilized.

When examining the histology of sperm retrieval specimens, the pattern seen can often be suggestive of the production of sperm. It is important to consider whether the histologic pattern analyzed was the most advanced pattern of histology, or the predominant histologic pattern, as the two analyses can provide very different information regarding the patient or testis being described. It has been shown that men with just Sertoli cell-only syndrome (SCO) have a much lower rate of spermatozoa recovery when compared to men with predominantly hypospermatogenesis (HS) and maturation arrest (MA) [21]. Histopathologic specimen alone has been shown to be the strongest single predictor of successful sperm retrieval with conventional techniques [59]. In micro-TESE performed on patients with Klinefelter syndrome, no single preoperative predictive variable was noted to predict a successful outcome, but there were several variables where an association was noted. Men with Sertoli cell-only on diagnostic biopsy had a sperm retrieval rate of 70%, and the presence of seminiferous tubules that did not have sclerotic changes during the procedure was associated with the most favorable outcomes [60].

Because histopathology is now usually examined at the time of sperm retrieval and is not typically a preoperative variable, it can be used to determine success of sperm retrieval only if a diagnostic biopsy was performed. One such way would be the use of testicular biopsies before a more definitive sperm retrieval is performed. The use of histopathology on testicular biopsy has been shown to have some benefit, with the predictive value of biopsy approaching 90% if a spermatozoon is seen when examining the biopsy specimen [61]. The finding of mature spermatozoa upon examination of the histopathologic specimen provides the greatest positive predictor for success of sperm retrieval [62]. Thus, testicular biopsy findings may provide the most accurate predictor of successful sperm retrieval, when positive, but of limited value – the vast majority of cases – when negative [35].

Despite this promising predictive value of biopsy findings, the performance of a biopsy on all patients before undergoing a sperm retrieval procedure is not necessarily desirable. The biopsy itself is a procedure with varied complications including post-procedural pain, infection, devascularization and bleeding leading to hematoma formation. Along with this, many patients will still go on to have a sperm retrieval procedure despite the biopsy findings unless it is a planned therapeutic biopsy.

## Conclusions

There is no individual characteristic or variable that can accurately predict the ability to retrieve sperm during a surgical procedure. While preoperative variables still do not provide a perfect model to predict success of micro-TESE, current research suggests that a combination of these variables can be used to counsel patients and help guide clinical decisions. More genetic, molecular and imaging studies are needed specifically on the prediction of outcomes of sperm retrieval in men with NOA to determine the most efficient way to guide clinicians when counseling their patients on the probability of success with micro-TESE.

## Abbreviations

NOA: Non-obstructive azoospermia
Micro-TESE: Microdissection testicular sperm extraction
ICSI: Intra-cytoplasmic sperm injection
TESE: Testicular sperm extraction
FSH: Follicle-stimulating hormone
KS: Klinefelter syndrome
hCG: Human chorionic gonadotropin
SRR: Sperm retrieval rate
SCO: Sertoli cell-only
HS: Hypospermatogenesis
MA: Maturation arrest.
